# Olfactory genes affect major depression in highly educated, emotionally stable, lean women: a bridge between animal models and precision medicine

**DOI:** 10.1038/s41398-024-02867-2

**Published:** 2024-04-08

**Authors:** Nora Eszlari, Gabor Hullam, Zsofia Gal, Dora Torok, Tamas Nagy, Andras Millinghoffer, Daniel Baksa, Xenia Gonda, Peter Antal, Gyorgy Bagdy, Gabriella Juhasz

**Affiliations:** 1 Department of Pharmacodynamics, Faculty of Pharmaceutical Sciences, Semmelweis University, Budapest, Hungary; 2 NAP3.0-SE Neuropsychopharmacology Research Group, Hungarian Brain Research Program, Semmelweis University, Budapest, Hungary; 3 Department of Measurement and Information Systems, Budapest University of Technology and Economics, Budapest, Hungary; 4 Department of Personality and Clinical Psychology, Institute of Psychology, Faculty of Humanities and Social Sciences, Pazmany Peter Catholic University, Budapest, Hungary; 5 Department of Psychiatry and Psychotherapy, Semmelweis University, Budapest, Hungary

**Keywords:** Depression, Personalized medicine, Human behaviour

## Abstract

Most current approaches to establish subgroups of depressed patients for precision medicine aim to rely on biomarkers that require highly specialized assessment. Our present aim was to stratify participants of the UK Biobank cohort based on three readily measurable common independent risk factors, and to investigate depression genomics in each group to discover common and separate biological etiology. Two-step cluster analysis was run separately in males (*n* = 149,879) and females (*n* = 174,572), with neuroticism (a tendency to experience negative emotions), body fat percentage, and years spent in education as input variables. Genome-wide association analyses were implemented within each of the resulting clusters, for the lifetime occurrence of either a depressive episode or recurrent depressive disorder as the outcome. Variant-based, gene-based, gene set-based, and tissue-specific gene expression test were applied. Phenotypically distinct clusters with high genetic intercorrelations in depression genomics were found. A two-cluster solution was the best model in each sex with some differences including the less important role of neuroticism in males. In females, in case of a protective pattern of low neuroticism, low body fat percentage, and high level of education, depression was associated with pathways related to olfactory function. While also in females but in a risk pattern of high neuroticism, high body fat percentage, and less years spent in education, depression showed association with complement system genes. Our results, on one hand, indicate that alteration of olfactory pathways, that can be paralleled to the well-known rodent depression models of olfactory bulbectomy, might be a novel target towards precision psychiatry in females with less other risk factors for depression. On the other hand, our results in multi-risk females may provide a special case of immunometabolic depression.

## Introduction

Major depressive disorder (MDD) is a common and heavily debilitating psychiatric condition [1], with high rates of treatment resistance, which poses an urgent need for the identification of reliable biomarkers that characterize distinct subgroups of patients with distinct therapeutic needs [2, 3]. Although this strategy, namely precision medicine, seems a promising approach in MDD therapy, present stratification strategies mainly use genetic, brain imaging or electrophysiological biomarkers as the starting point [4–6], all of which biomarkers are of high cost and requiring specific equipment to assess.

A novel approach to understand the heterogeneous genetic underpinnings of depression could come from first stratifying patients according to various, more easily and efficiently measurable, established risk factors or endophenotypes of MDD, which could even aggregate into distinct profiles or constellations to form data-driven, solid patient subgroups, followed by exploring unique and specific biomarkers for each identified subgroup. Because of the easy measurement of clustering variables in the first step, successfully identified biomarkers in the second step could pave the way towards tailored interventions or even prevention methods which are feasible in the everyday practice of general practitioners or mental health providers.

In order to stratify subjects along this novel approach, three common, well-established, and easily assessed risk factors of MDD offer a good choice: neuroticism, body fat percentage, and years spent in education. Neuroticism is a personality trait, representing a stable tendency to experience negative emotions, and its high level is a firm endophenotype for depression, meaning that it lies on the causal pathway between genes and the disorder [7, 8]. High body fat percentage [9–11] as an internal, and low level of education as an external risk factor [8, 12–14] have also been robustly associated with MDD risk. Moreover, depressogenic effects of these three risk factors are also dependent on each other: the constellation of neuroticism and body fat percentage [15], or of neuroticism and socio-economic status [15, 16] has been reported to be associated with depression severity. Another important moderating variable is sex: the depressogenic effect of high body fat percentage was more pronounced in women than in men [11], and the depressogenic effect of low education level was only present in women in a study [17]. Moreover, effects of polymorphisms within the serotonin transporter gene on neuroticism and depression were also dependent on sex, being the homozygotic low-expression genotype a risk only in men but not in women [18].

Our present aim was to reveal the genomic background of MDD within separate subgroups that can be characterized by distinct risk or protective patterns of sex, neuroticism, body fat percentage, and years spent in education. Such a stratification in genomics may then lead to the discovery of novel drug targets or biomarkers.

## Materials and methods

### Participants

Under application number 1602, we analyzed data from white British UK Biobank participants who had provided a written informed consent. Invitation and recruitment were based on NHS patient registers of people aged 40–69 years [19]. Ethical approval was given by the National Research Ethics Service Committee North West–Haydock [20], and all procedures were carried out in accordance with the Declaration of Helsinki.

In our present study, we included participants who passed genomic quality control (QC) and filtered out those who had missing data on any of the variables of sex, neuroticism score, body fat percentage, years in education, age, genotyping array, lifetime depression, and current depression score. These filtering steps yielded 149,879 males and 174,572 females in our present analyses. Age ranged between 39 and 72 years in males, and between 40 and 71 years in females.

### Phenotypes

Neuroticism score was calculated as the sum of 12 dichotomous items of the EPIN-R questionnaire [19], divided by the number of responded items for each participant, then multiplied by 12, yielding a range between 0 and 12.

Body fat percentage (data field ‘23099’ in UK Biobank) was calculated based on impedance measurement and body composition estimation. Body fat percentage is considered high above 25% in males and above 35% in females [15].

Years spent in education was derived from data field ‘6138’, based on the recoding system by [21] and [22], and yielding a range between 7 and 20 years of education.

Lifetime depression status was based on a lifetime diagnosis of either a depressive episode (F32 according to ICD-10, data field ‘130894’) or recurrent depressive disorder (ICD-10 F33, data field ‘130896’).

Current depression score was calculated as the sum of four depression items detailed in [15] and [23], divided by the number of responded items and multiplied by four, yielding a range between 4 and 16.

Mean and standard error of mean of each continuous variable is presented in Supplementary Table 1.

The prerequisite of cluster analyses is that clustering variables should be relatively independent of each other, being thus capable of showing various patterns or profiles in distinct subgroups of participants. Neuroticism, body fat percentage (henceforth body fat), and years spent in education (henceforth education) showed significant and low Pearson correlation values with each other (Supplementary Table 2), with the maximum absolute values of correlation between body fat and education, which were −0.149 in males and −0.147 in females.

### Genotyping, imputation, and genomic quality control

QC procedure of UK Biobank, as well as our additional QC steps for variants and participants were implemented as detailed in [23], with the only exception that in this study we involved X and Y chromosomes, as well as pseudo-autosomal regions of X and Y chromosomes, in addition to autosomal chromosomes. Single-nucleotide polymorphisms (SNPs) and genes were positioned according to the GRCh37/hg19 genome assembly.

### Analyses

A priori cluster analyses demonstrated that including sex as a clustering variable in addition to neuroticism, body fat, and education would result in two clusters of male and female, but both clusters would be excessively heterogeneous internally (average Silhouette coefficient for the model was 0.4). Consequently, we ran the same cluster analysis model separately in males and females, with neuroticism, body fat, and education as clustering variables.

Two-step cluster analysis was run using IBM SPSS 29, with default settings of cluster features tree tuning and without outlier noise handling in the first step. In the second step, for the agglomerative clustering, the optimal cluster number was determined by Schwarz’s Bayesian Information Criterion (BIC) with a maximum of 15. All three variables were standardized for the analysis, and a log-likelihood distance measure was used. An average Silhouette coefficient above 0.5 indicates a good solution, with clusters that are internally homogeneous and at the same time distant from all the other clusters. To ensure the stability of the clustering solution, the same cluster analysis method was repeated 20 times in each sex, with 20 distinct random orders of the participants. From the 20 resulting solutions, we chose the one that yields a total correlation of cluster membership with another solution out of the 20, and at the same time has the highest possible Silhouette coefficient. From these two highly correlating models, in each sex we chose the one that would yield a larger sample size in the smaller cluster.

To compare clusters with each other, descriptive statistics were also done using IBM SPSS 29.

For SNP-level genome-wide association study (GWAS) within each cluster, Plink2 [24] was used (accessed on 8–10 February 2024). To control for population stratification, top ten principal components (PCs) of the genome were calculated with an approximative method [25] within each of the four clusters (see below, in *Cluster analyses and description of the clusters* part of *Results* section). Logistic regression models were run for lifetime depression status as outcome, with each SNP, age, genotyping array, and the top ten PCs as predictors. In males 6,266,283 SNPs and in females 6,266,189 SNPs survived genomic QC, which, together with the two clusters in each sex, entailed a *p* < 1.9948 × 10^−9^ Bonferroni-corrected significance threshold. Continuous variables were standardized in the analyses. SNP-level GWAS results were entered into further analyses.

The Complex-Traits Genetics Virtual Lab (CTG-VL) web platform [26] was used for SNP heritability, genetic correlation, and latent causal variable (LCV) analyses (accessed on 13 February 2024). Within all these analyses, the European reference population of the 1000 Genomes (phase 3) database was used for linkage disequilibrium (LD) calculation.

The LD Score Regression (LDSC) method calculates LD score for each SNP, which means the amount of genetic variation tagged by the SNP. LD score is calculated as the sum of all R^2^ scores measured with other SNPs, and has a between-SNP variability because of LD structure of SNPs. Then, the method uses this LD score variable as a predictor in a regression model for GWAS test statistics as outcome, thus disentangling genuine polygenic association effects from other confounding effects that can inflate GWAS test statistics [27]. LDSC method was used for SNP heritability, genetic correlation, and LCV analyses.

The LCV model hypothesizes a latent variable behind the genetic correlation of two GWAS-s, and calculates genetic correlation of each GWAS with this latent variable. It introduces the concept of “genetically causal” reflecting the asymmetry of the effect of this latent variable on the two GWAS-s [28]. Using this term, the GWAS that can be better explained by the latent variable is partially “genetically causal”, and the other GWAS goes beyond the latent variable and has more unique variance. Genetic causality proportion (GCP) ranges from 0 to 1 in absolute values, with an absolute value > 0.6 denoting a high level of genetic causality. For both genetic correlation and LCV analyses, our six tests yielded a *p* < 0.0083 significance threshold.

FUMA v1.5.2 (accessed on 13 February 2024) was used for MAGMA v1.08 [29] and GENE2FUNC analyses [30].

MAGMA gene-level analyses used position, with an extended gene boundary of ±10 kilobase [31], to map SNPs to protein-coding genes having Ensembl ID. Gene-level *p*-values were computed with a SNP-wise mean model, and then were probit-transformed into Z-scores to be entered into the other two MAGMA analyses, with a high Z-score meaning a low *p*-value. 19,868 genes in the four clusters resulted in a *p* < 6.2915 × 10^−7^ corrected significance threshold.

MAGMA gene set-level analyses regressed gene Z-score against gene set membership, including gene size, minor allele count, LD between SNPs, and between close genes as additional predictors. A separate model was run with each gene set, and all gene sets of MsigDB v7.0’s C2 (curated) and C5 (Gene Ontology—GO) gene set collections were tested. 17,012 gene sets in the four clusters resulted in a *p* < 7.3478 × 10^−7^ corrected significance threshold.

MAGMA tissue-specific gene expression analyses regressed gene Z-score against gene expression level within a specific tissue, including technical confounders and the average expression across all tissues as additional predictors. This test is one-sided, hypothesizing a positive association between gene Z-score and tissue-specific gene expression. Tested tissues included the 11 brain developmental stages of BrainSpan database [32], which, together with the four clusters, entailed a *p* < 0.0011 significance threshold.

For additional FUMA analyses, genomic risk loci were defined with a *p* ≤ 1 × 10^−5^ for lead SNPs, and criteria of *p* ≤ 0.05 and *R*^2^ ≥ 0.6 with the lead SNP, for other SNPs to be included in the genomic risk locus. A minor allele frequency (MAF) ≥ 0.01 criterion was also applied for all SNPs. The European reference population of the 1000 Genomes (phase 3) database was used for calculation of *R*^2^ and MAF, and for involving additional SNPs into the loci that are not present in our database. All types of Ensembl v110 genes were mapped to SNPs of genomic risk loci if any of the following criteria was met. First, as in MAGMA analyses, if the SNP is located within gene boundaries extended by ±10 kilobase. Second, if the SNP-gene pair is significant at a false discovery rate (FDR) ≤ 0.05 as an expression quantitative trait locus (eQTL) within any of the available brain tissues or cell types (see Supplementary File 1). Third, if regions of the SNP and the gene’s promoter (defined as 250 base-pair upstream and 500 base-pair downstream of the transcription start site) are in a chromatin interaction loop with each other at an FDR ≤ 1 × 10^−6^ level within any of the available brain tissues or cell types (see Supplementary File 1). Genes thus mapped to genomic risk loci’s SNPs by any of these three methods were then tested with GENE2FUNC hypergeometric test for enrichment in each of the MsigDB v7.0 C2 and C5 gene sets (detailed above). Benjamini-Hochberg FDR correction was used within each gene set category (these are: C2 curated gene sets; C5 GO biological process; C5 GO cellular component; and C5 GO molecular function), and the adjusted *p*-value cutoff was set to *p* < 0.0125 because of the four clusters. Among significantly enriched gene sets, those with the highest proportions of our mapped genes were interpreted.

## Results

### Cluster analyses and description of the clusters

Cluster analyses based on three common and well-established MDD risk factors revealed a two-cluster solution as the best in both the male and female groups, with a Silhouette coefficient of 0.6183 in males, and 0.6076 in females. Both values indicate that neuroticism, body fat, and education, when considered simultaneously, can compose patient subgroups (strata) that are internally homogeneous but markedly distinguishable from each other.

**Fig. 1:**
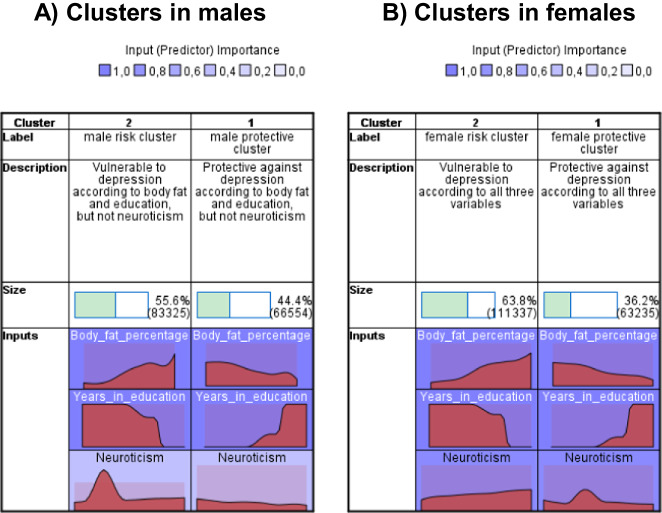
Clusters based on neuroticism, body fat percentage, and years spent in education, separately in males and females. **A** Clusters in males; **B** clusters in females. Clusters sizes and descriptions are shown, as well as input variables’ relative distribution in each cluster within sex, and their importance in clustering in each sex. In females, two solid clusters can be seen: in one of them, all three input variables show a risk for depression (female risk cluster), and in the other one all three variables point to a protective direction against depression (female protective cluster). However, in males, neuroticism shows a protective direction against depression not in the protective cluster but in case of a risk pattern of body fat percentage and education (male risk cluster). Nevertheless, neuroticism proved a less important input variable in clustering in males than any other variable in either sex.

However, the distribution patterns of input variables in the two clusters show some differences between the two sexes, particularly in case of neuroticism (Fig. 1). Neuroticism proved to be somewhat less important in clustering within males than within females, and somewhat less important in males than any other input variable in either sex (Fig. 1). Relative frequencies of the specific values of the input variables in each cluster are shown in Supplementary Figs. 1 and 2.

As it could be expected, lifetime depression (depressive episode or recurrent depressive disorder) diagnosis was significantly more frequent in the male risk cluster (8.8%) than in the male protective cluster (7.4%), and significantly more frequent in the female risk cluster (13.3%) than in the female protective cluster (11.8%) (both Fisher exact test’s *p* < 0.001). Other descriptive statistics for the clusters are detailed in Supplementary File 1.

Our genome-wide SNPs explain around 2–4% of the variability in lifetime depression status within each cluster (Table 1). Intercept of the regression of GWAS test statistics against LD score is below 1.10 in each cluster, suggesting that our SNP-level genetic associations are genuine [33]. An inflation ratio is calculated from this intercept, and it measures the proportion of test statistics’ inflation that can be attributed to causes other than polygenic heritability. It is expected to be close to zero, but it can be even 10–20% because of LD score mismatch between sample and reference, or because of low LD-scored SNPs having larger effect on the outcome [27]. Although standard errors of ratio are high in each cluster, the expected value of the inflation ratio is exceeded only in the male protective cluster (Table 1).

**Table 1:** SNP heritability (variability of lifetime depression status accounted for by the whole set of SNPs) within each cluster.

Cluster	SNP *h*^2^	Lower 95% CI of SNP *h*^2^	Upper 95% CI of SNP *h*^2^	Intercept (SE)	Ratio (SE)
male risk cluster	0.0252	0.013832	0.036568	0.9974 (0.0068)	<0
male protective cluster	0.022	0.006516	0.037484	1.01 (0.0069)	0.2491 (0.1704)
female risk cluster	0.0277	0.01888	0.03652	1.0029 (0.0069)	0.0432 (0.1035)
female protective cluster	0.0329	0.018788	0.047012	1.0035 (0.0063)	0.0768 (0.1386)

Intercept and ratio from the LD Score Regression model are also shown. The expected value for intercept is <1.10; and for ratio is a maximum of 10–20%.

*SNP* single-nucleotide polymorphism, *h*^*2*^ heritability, *CI* confidence interval, *SE* standard error, *LD* linkage disequilibrium.

### Overlap between the clusters in depression genomics

Table 2 shows that all clusters have a high genetic intercorrelation in depression genomics with each other, although these genetic correlation values have relatively high standard errors.

**Table 2:**
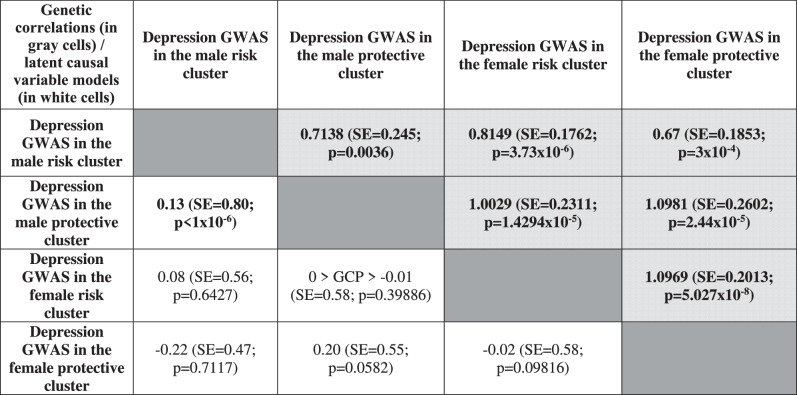
Genetic correlations (in gray cells) and latent causal variable model’s GCP results (in white cells), in depression GWAS-s between each pair of clusters.

Significant results are marked with bold. Latent causal variable model hypothesizes a latent variable behind the genetic correlation of two GWAS-s, and tests the asymmetry in the effect of this latent variable on the two GWAS-s (“genetic causality”). The GWAS we assume to be “genetically causal” represents the latent variable (i.e., the genetic variance shared between the two GWAS-s) more, while the other GWAS has relatively more unique genetic variance. Positive direction of GCP refers to the GWAS in the given row as “genetically causal”, and negative direction refers to the GWAS in the given column as “genetically causal”. |GCP| ranges 0–1, with an absolute value > 0.6 denoting a high level of genetic causality.

*GCP* genetic causality proportion; *GWAS* genome-wide association study; *SE* standard error.

However, these high genetic intercorrelations cannot be explained by asymmetries in genetic overlap. Particularly, only the two male clusters showed a significant GCP value with each other, but it was not high enough to suggest genetic causality (Table 2, direction of GCP refers to the particular cluster which has more unique genetic risk for depression, compared to the other cluster).

### Unique genetic hits for depression in the clusters

No significant hit emerged in any of the clusters, at SNP level, MAGMA gene level (Supplementary Tables 3–6), MAGMA gene set level (Supplementary Tables 7–10), or in MAGMA tissue-specific gene expression (Supplementary Tables 11–14).

However, FUMA GENE2FUNC analyses, which take the advantage of eQTL mapping and chromatin interaction mapping in addition to positional mapping of genes to SNPs, revealed significant hits that survived correction. In the male risk cluster, the *Johnstone_parvb_targets_2_dn* gene set of the C2 curated gene set collection showed an adjusted *p*-value of 0.00696. Nevertheless, since this gene set is defined in breast cancer cells, this result will not be discussed in the present paper.

**Fig. 2:**
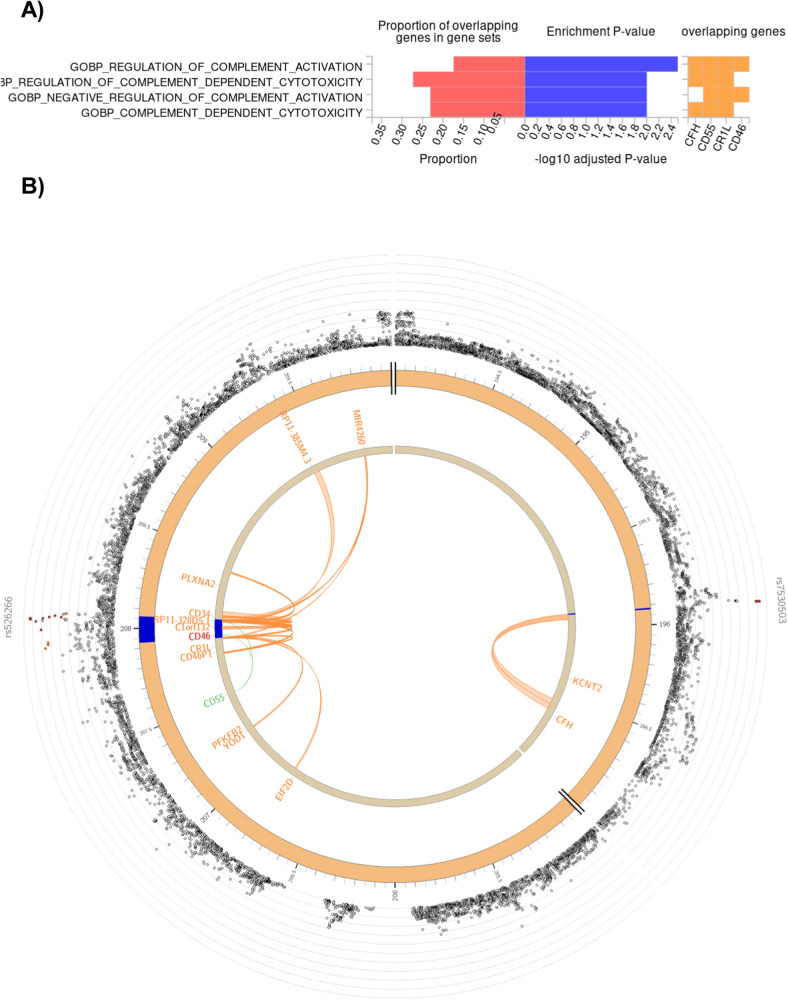
Significant enrichment of genes mapped to SNPs of depression risk loci of the female risk cluster, in gene sets of C5 GO BP; and genes mapped by eQTL association (green), chromatin interaction (orange), or by both (red), to this cluster’s depression risk loci (blue) on chromosome 1. **A** Our mapped genes, as well as their proportion within each gene set, and *p*-value of their enrichment in each gene set are shown. Benjamini–Hochberg FDR correction was applied within each gene set category, and the adjusted *p-*value cutoff was *p* < 0.0125 because of the four clusters. Only results surviving correction are shown. **B** Top SNP of each risk locus is named, and all SNPs are shown as dots on a −log_10_*p-*value axis and in their chromosomal position, with red dots representing an *R*^2^ > 0.8 and orange dots an *R*^2^ > 0.6 linkage with the top SNP. *CFH*, *CD55*, *CR1L*, and *CD46* are the mapped genes from two distinct loci that show a significant enrichment in complement gene sets. *SNP* single-nucleotide polymorphism, *GO* Gene Ontology, *BP* biological process, *eQTL* expression quantitative trait locus, *FDR* false discovery rate.

In the female risk cluster, genes mapped to depression risk loci significantly enriched in complement-related GO BP gene sets, from which the most represented were *GO BP regulation of complement-dependent cytotoxicity* (proportion of our mapped genes within: 0.2727), *GO BP negative regulation of complement activation*, and *GO BP complement-dependent cytotoxicity* (both having a 0.23077 proportion of our mapped genes) (Fig. 2A). Our mapped genes of these gene sets reside in two distinct loci within chromosome 1 (Fig. 2B), conveying a reliability to our results.

**Fig. 3:**
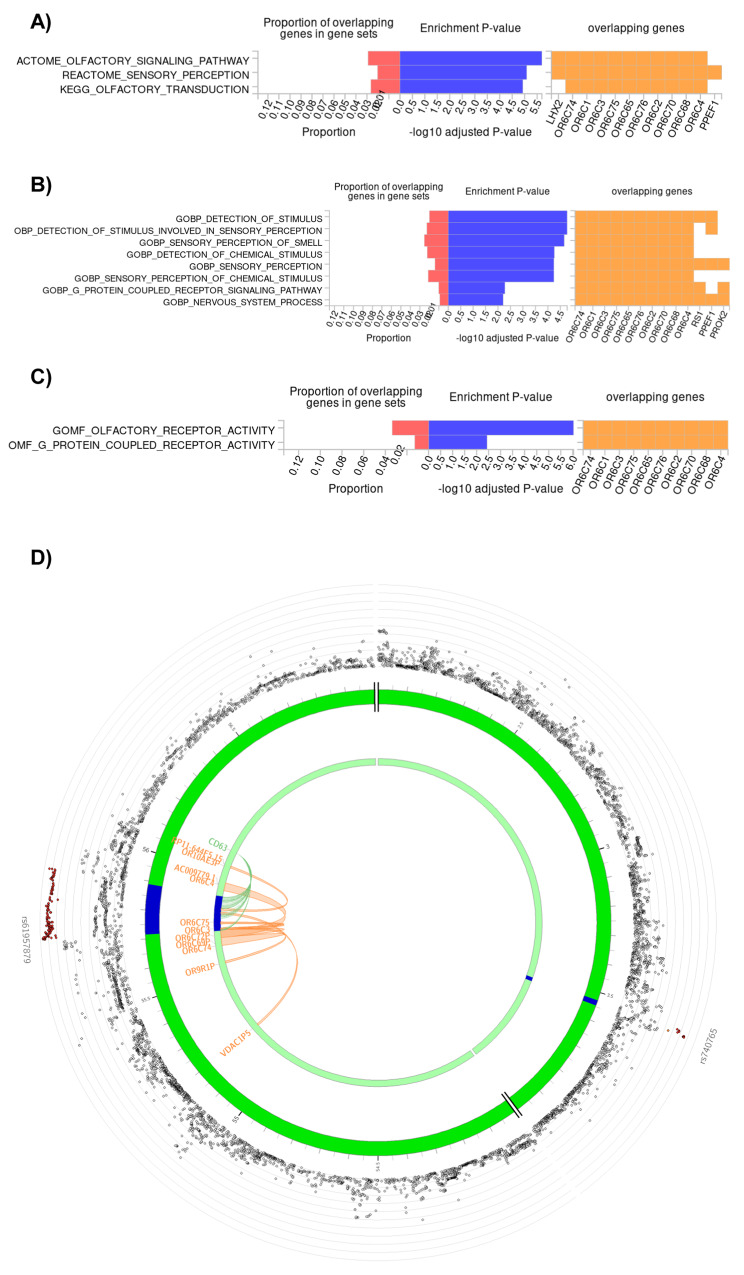
Significant enrichment of genes mapped to SNPs of depression risk loci of the female protective cluster, in gene sets of C2 curated, C5 GO BP, and C5 GO MF gene set collections; and genes mapped by either eQTL association (green) or chromatin interaction (orange), to this cluster’s depression risk loci (blue) on chromosome 12. **A**–**C** Our mapped genes, as well as their proportion within each gene set, and *p-*value of their enrichment in each gene set are shown. Benjamini–Hochberg FDR correction was applied within each gene set category, and the adjusted *p-*value cutoff was *p* < 0.0125 because of the four clusters. Only results surviving correction are shown. **D** Top SNP of each risk locus is named, and all SNPs are shown as dots on a −log_10_*p-*value axis and in their chromosomal position, with red dots representing an *R*^2^ > 0.8 and orange dots an *R*^2^ > 0.6 linkage with the top SNP. *OR6C74*, *OR6C3*, *OR6C75*, and *OR6C4* are the mapped odorant receptor genes that show a significant enrichment in olfactory gene sets. *SNP* single-nucleotide polymorphism, *GO* Gene Ontology, *BP* biological process, *MF* molecular function, *eQTL* expression quantitative trait locus, *FDR* false discovery rate.

Genes mapped to depression risk loci in the female protective cluster significantly enriched in olfactory gene sets, from which *GO MF olfactory receptor activity* and *Reactome olfactory signaling pathway* were the most represented by our mapped genes, with proportions of 0.03367 and 0.029, respectively (Fig. 3A–C). All but one mapped genes of these gene sets encode odorant receptors, and are located in the same locus of chromosome 12 (Fig. 3D). The only exception is the transcriptional activator gene *LHX2* located in another part of the genome, on chromosome 9, strengthening the reliability of our result.

## Discussion

Despite high genetic intercorrelations between the specific clusters in depression genomics, as well as no detectable asymmetry in this genetic overlap between the clusters, the two female clusters have shown unique genetic results. Complement system genes emerged in the background of depression of the female cluster with a risky pattern of high neuroticism, high body fat, and low education, suggesting a possible pathophysiological role of inflammation and this specific part of the immune system within this well-characterized female subgroup. On the other hand, olfactory genes enriched in the background of depression in the female cluster having a protective constellation of low neuroticism, low body fat, and high education, pointing to a specific relevance of rodent and human olfactory models of depression within this specific subgroup.

Our findings that depression has sex-dependent unique genetic background was suggested by previous studies. For example, depression has been associated in women’s brain with downregulated immune-related, oligodendrocyte-related and microglia-related genes but upregulated synapse-related genes, while in men, an opposite pattern of upregulated oligodendrocyte and microglia genes as well as downregulated synapse genes were observable in MDD compared to controls [34]. The two sexes have also shown some differences in the relative importance of biological pathways behind MDD: in women’s brain transcriptomics, MAPK activity, inflammatory response, and synaptic transmission had the highest ranks, while in men’s brain, transmission of nerve impulse, organic acid metabolism, and catecholamine metabolism regulation mattered best in MDD [35]. However, our present results suggest that in addition to sex-dependent biological pathways, there are some other pathways behind depression, e.g., olfaction, that may depend not only on sex but on its constellation with other moderating factors. These additional moderating factors may also refine some former sex-dependent results, such as inflammatory response and immune system functioning into complement system, in the case of a specific subgroup of women. Nevertheless, our study also suggests with regard to depression genomics that different constellations of neuroticism, body fat, and education seem useful in the further stratification only of women but not of men.

### Clusters are distinct based on the pattern of input variables, but are quite similar in terms of depression genomics

Neuroticism as an endophenotype, body fat percentage as an internal, and education level as an external risk factor for depression, in addition to sex, seem appropriate variables to define phenotypically distinct but internally homogeneous subgroups (strata) of the general adult (middle-aged and elderly) British population. Despite the phenotypic differences, these strata cannot be apparently distinguished by the genomic background of MDD.

Compared to our results, SNP-based heritability of MDD has been suggested to be somewhat higher, around 5–6%, in the whole UK Biobank [36] or another population sample [37], and much higher, 19–21%, if enrolling severe MDD participants [38] or MDD patients with atypical symptoms of hypersomnia and weight gain [36]. However, our depression phenotype was composed of the lifetime occurrence of either a single episode or a recurrent disorder, thus combining typical and atypical depression cases of any severity, which may explain these discrepancies, although convey the widest perspective of further applicability to our results.

### Complement system in a refinement of the immunometabolic depression concept, for the special case of multi-risk women

A conceptual model of immunometabolic depression has been proposed on the association of depression with immunometabolic dysregulations, also highlighting the role of depression heterogeneity in these associations [10]. The model has already emphasized the impact of obesity and low socio-economic status [10, 39], but our results now can add the neuroticism endophenotype as a further moderating factor in depression heterogeneity. Moreover, our results with complement system genes suggest one possible way to refine the concept of immunometabolic depression, particularly for the special case of females at risk for depression according to three well-characterized factors: body fat, education, and neuroticism.

Different complement activation pathways converge on C3 protein, which has shown a higher mRNA level in the prefrontal cortex of depressed suicide victims compared to controls [40]. In line with this result, C3 knock-out mice were resistant to depressive-like behavior induced by the chronic unpredictable mild stress (CUMS) model [40]. Driven by the potential role of complement system in synaptic pruning within the central nervous system, a recent study investigated plasma levels of seven complement proteins in MDD patients and controls, and found levels of C1q, complement factor B, and complement factor H (CFH) higher in MDD compared to controls [41]. *CFH* was among the genes mapped in brain tissues to depression risk SNPs in our female risk cluster, and also has shown a genetic association with MDD in a former study [42]. A plasma CFH level higher in MDD compared to controls has been corroborated by other studies [43, 44], with some results pointing to the association of high plasma CFH level with anhedonia [44, 45], although CFH plasma level was lower in MDD compared to controls in a Han Chinese sample [42]. It is important to note that all these studies with CFH had a female predominance in their MDD samples [41–43, 45], even if slightly [44], which is in line with our results specific to a female cluster.

### Olfactory genes as MDD biomarkers that are specific for the female protective cluster

Only in females with low levels of neuroticism and body fat percentage but with many years spent in education, odorant receptor genes emerged as a potential unique biomarker of MDD.

MDD has been linked to multiple stages of olfactory perception, from periphery to cortex. Projections from the entorhinal cortex to the hippocampus [46] and visual cortex [47] may play a role in the pathogenesis of depression. Alternatively, the association of olfactory functions with depression may be due to their association with emotional processing in general, underpinned by a study that demonstrated lower right hippocampal brain responses to emotional pictures in patients with acquired olfactory loss compared to controls, independently of depressive symptoms [48]. Olfactory bulbectomy is a rodent model of depression, entailing perturbations in the frontal cortex that are similar to those of human MDD [49]. In addition, olfactory bulb dysfunction in rats can be induced by the CUMS model, which deterioration is mediated by mechanisms of neurogenesis, energy metabolism [50], purine and lipid metabolism [51]. Regarding human MDD, reduced volume parameters of the olfactory bulb [52] and olfactory sulcus [53] are suggested to be stable trait markers of MDD, deteriorating response to psychotherapy, and worsening residual symptoms, respectively. In contrast, odor identification performance, which is specifically impaired in mood disorders but not in anxious patients [54], will improve after remission of MDD [55, 56]. Although olfactory dysfunctions in MDD can be as well attributed to impaired feedback mechanisms of reward systems [56], or to anhedonia [57], our GWAS hit with odorant receptor genes underpins the ‘bottom-up’ way of depression pathogenesis as opposed to the ‘top-down’ way. This ‘bottom-up’ way is also reflected in that the depressogenic CUMS intervention in rats damages olfactory epithelium and olfactory receptor neurons as well [56]. However, ‘bottom-up’ ways can also be permanently modulated by ‘top-down’ ways, since a reduced turnover rate of olfactory receptor cells in depression can also be due to a reduced attention to odors, and thus an enhancement of awareness to odors by a “smell training” may improve depression via improvement of attention to odors [58]. Moreover, unilateral “smell training” in healthy participants has been shown to increase olfactory bulb volume on the contralateral side as well, while worsening odor thresholds [59]. All these former results may converge to putative novel therapeutical or even prevention possibilities in a well-characterized non-risk subgroup of women.

Our results are specific for only one of the four clusters, which is in line with the former inconclusive associations between depression and olfactory dysfunctions [58]. Our results suggest that in females, in the absence of well-established depression risk associated with high body fat percentage or low education level, MDD does not emerge via the causal pathway of the neuroticism endophenotype, but may be associated with olfactory genes. Neuroticism has been suggested to be an equifinal endpoint of several critical developmental periods [60] and biological pathways [33], all of which may have different weights in each individual, therefore they may mask the effect of each other on the way to depression. In contrast, we may see more primary or elementary mechanisms in depression genetics in case of emotional stability and in the simultaneous lack of other classical risk factors.

### Limitations

Our study has some limitations to consider. First, FUMA GENE2FUNC hypergeometric test does not correct for LD between close genes. However, our mapped complement and olfactory genes are both from two distinct genomic loci, conveying reliability to our results in both female clusters. Moreover, our GENE2FUNC results for the female protective cluster are also corroborated by MAGMA gene-level results, which, although not surviving correction for multiple testing, yield *OR6C1* and *OR6C3* at a 10^−6^ *p*-level, *OR6C75*, *OR6C76* at a 10^−5^ *p*-level, and *OR6C70*, *OR6C65*, and *OR6C2* at a 10^−4^ *p*-level (all of them are mapped in GENE2FUNC), and provide further odorant receptor genes, *OR6C6* from the same locus of chromosome 12, as well as *OR1J4*, *OR1N1*, and *OR1J2* from another locus on chromosome 9, all at a 10^−4^ *p*-level (Supplementary Table 6). MAGMA gene-based tests used a SNP-wise mean model, which is more sensitive to the mean SNP association within a gene, somewhat compensating the drawbacks of GENE2FUNC tests.

Furthermore, the inflation ratio value of MDD’s SNP heritability within the male protective cluster exceeds the desirable 20% [27], which suggests us to interpret results of this cluster with caution.

Moreover, study design of the UK Biobank is unable to explore real causal relationships behind the revealed association patterns. Consequently, our present results should only be used as descriptors of distinct subgroups in the future, but not regarded as firm causes of depression.

In addition, only participants with a white British ancestry (defined as self-report and genetic ancestry as well) were included because this is the subset that constitutes the majority of UK Biobank participants [61]. Future research should replicate our findings within population samples of different ancestries.

Finally, our SNP heritability results highlight the limited translatability of complement genes and olfactory genes into potential future precision medicine strategies. Particularly, only 2.77% and 3.29% of lifetime depression variability can be explained by the whole set of our SNPs in the female risk and protective cluster, respectively.

## Conclusions

We identified MDD genetic risk factors that are dependent simultaneously on sex, on an endophenotype, as well as on internal and external modifying risk factors of depression.

At the expense of relatively small variances explained by the whole set of genetic variants, our results can be more generalizable to either typical or atypical depression of any severity. We intended to conceptualize depression heterogeneity not at a clinical level (such as [10]) but only from a possible pathophysiological perspective. This perspective, along with a successful identification of three readily measurable putative moderating factors of depression genomics in females among the numerous moderators, can even be more useful in future depression prevention than considering clinical heterogeneity of the already manifested disorder.

In spite of a considerable genetic overlap between the phenotypically distinct clusters in depression genomics, the two female clusters yielded unique genetic results for depression. Reliability of complement system genes in the female risk cluster is corroborated by two distinct genomic loci. Moreover, reliability of olfactory genes in the female protective cluster is corroborated by two distinct genomic loci as well as highly significant gene-level results from another testing model.

After replication in populations of diverse genetic ancestries and/or socio-cultural background, our results can provide some contribution to novel precision medicine approaches in the prevention and maybe even therapy of MDD.

### Supplementary information

Supplementary File 1

Supplementary File 2

## Data Availability

The data presented in this study are available on request from the corresponding author. The data are not publicly available due to ethical considerations.
