# Supplementary material for: Olfactory genes affect major depression in highly educated, emotionally stable, lean women: a bridge between animal models and precision medicine
Source: Transl Psychiatry. 2024 Apr 8;14:182. doi: 10.1038/s41398-024-02867-2 (PMC11002013; doi:10.1038/s41398-024-02867-2)
Supplement: Supplementary file 1 [file 41398_2024_2867_MOESM1_ESM.docx]

**Materials and methods**

**Phenotypes**

***Supplementary Table 1.*** Mean and standard error of mean for continuous variables in the two sexes.

|  | **Males** | | **Females** | |
| --- | --- | --- | --- | --- |
|  | Mean | Standard error of mean | Mean | Standard error of mean |
| Neuroticism score (scoring 0-12) | 3.724 | 0.0084 | 4.723 | 0.0078 |
| Body fat percentage | 25.259 | 0.0149 | 36.561 | 0.0164 |
| Years in education (ranging 7-20) | 14.249 | 0.0134 | 13.580 | 0.0120 |
| Current depression score (scoring 4-16) | 5.449 | 0.0052 | 5.721 | 0.0051 |
| Age | 57.065 | 0.0209 | 56.617 | 0.0189 |

***Supplementary Table 2.*** Intercorrelations between clustering (input) variables separately in the two sexes. All correlations are significantly low.

|  | **Males** | | **Females** | |
| --- | --- | --- | --- | --- |
|  | Pearson correlation | p-value | Pearson correlation | p-value |
|  |  |  |  |  |
| Neuroticism - Body fat percentage | 0.008 | 0.002 | 0.021 | p<0.001 |
| Neuroticism - Years in education | -0.057 | p<0.001 | -0.092 | p<0.001 |
| Body fat percentage - Years in education | -0.149 | p<0.001 | -0.147 | p<0.001 |

**Analyses**

- **Databases used for expression quantitative trait locus (eQTL) SNP-gene mapping in FUMA (accessed on 13 February 2024)**:
  - eQTL catalogue BrainSeq brain
  - eQTL catalogue Schwartzentruber 2018 sensory neuron
  - PsychENCODE eQTLs: <http://resource.psychencode.org/>
  - xQTLServer: <https://mostafavilab.stat.ubc.ca/xqtl/>
  - CommonMind Consortium: <https://www.synapse.org//#!Synapse:syn5585484>
  - BRAINEAC: <http://www.braineac.org/>
  - GTEx v8 Brain & Pituitary: <https://www.gtexportal.org/home/datasets>
- **Databases used for chromatin interaction SNP-gene mapping in FUMA (accessed on 13 February 2024)**:
  - PsychENCODE EP links (one way): <https://www.ncbi.nlm.nih.gov/pmc/articles/PMC6413328/pdf/nihms-1015644.pdf>
  - PsychENCODE Promoter anchored loops: <https://www.ncbi.nlm.nih.gov/pmc/articles/PMC6413328/pdf/nihms-1015644.pdf>
  - Hi-C loops from Giusti-Rodriguez et al. 2019 („Pre-processed enhancer-promoter and promoter-promoter interactions based on HiC data for adult and fetal human brain samples”): adult cortex & fetal cortex
  - HiC(GSE87112) Dorsolateral prefrontal cortex & hippocampus & neural progenitor cell: <https://www.sciencedirect.com/science/article/pii/S2211124716314814?via%3Dihub>

**Results**

**Descriptive statistics for the clusters**

| **Male risk cluster** | **Male protective cluster** |
| --- | --- |
| 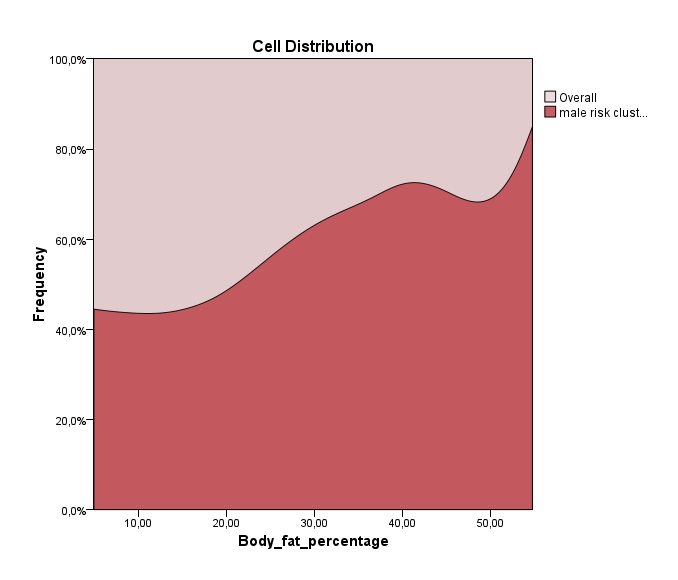 | 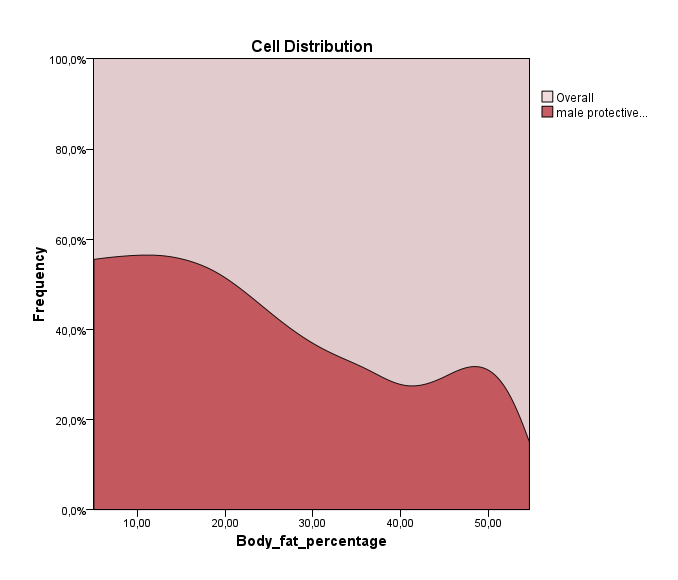 |
| 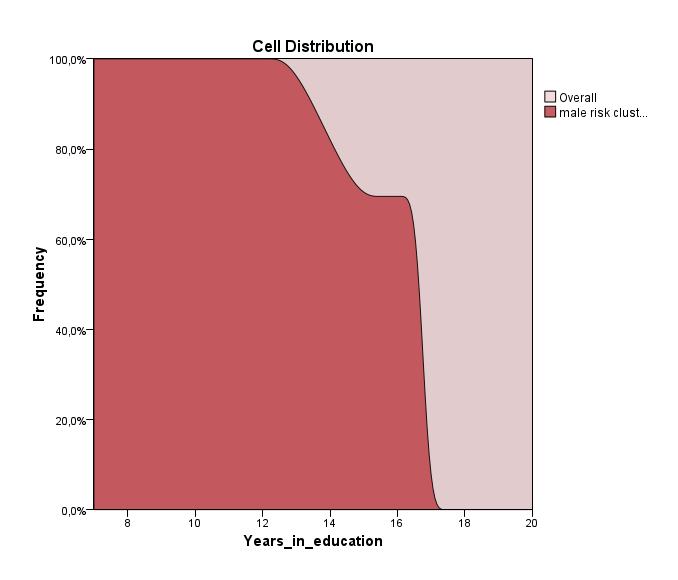 | 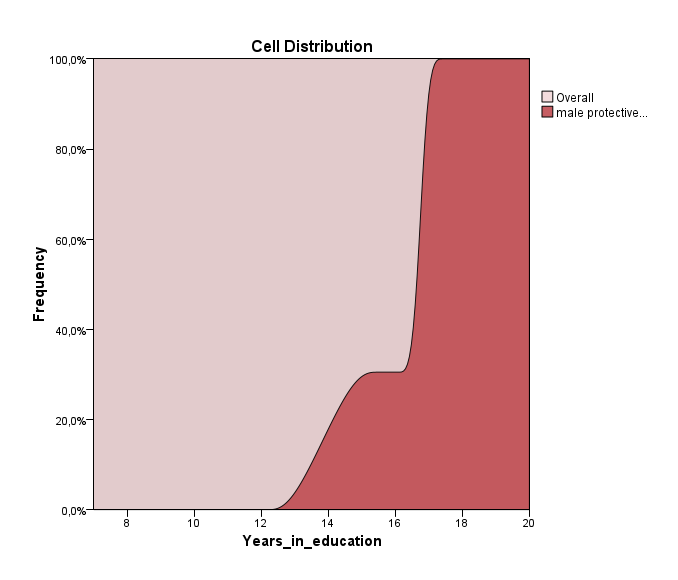 |
| 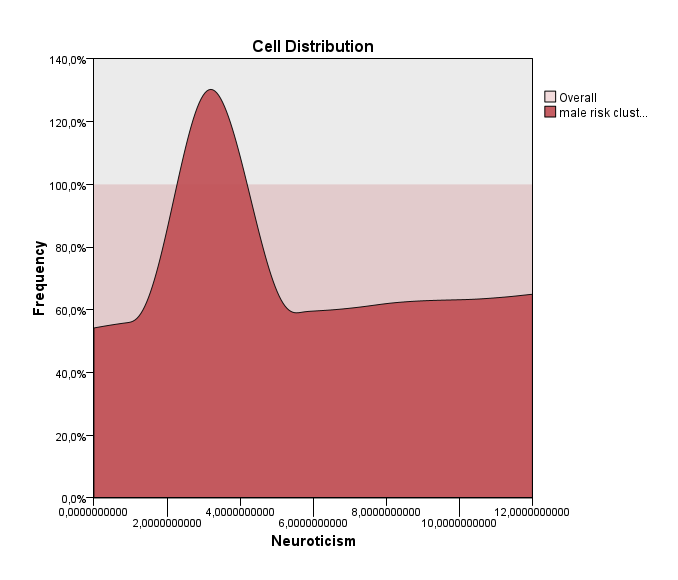 | 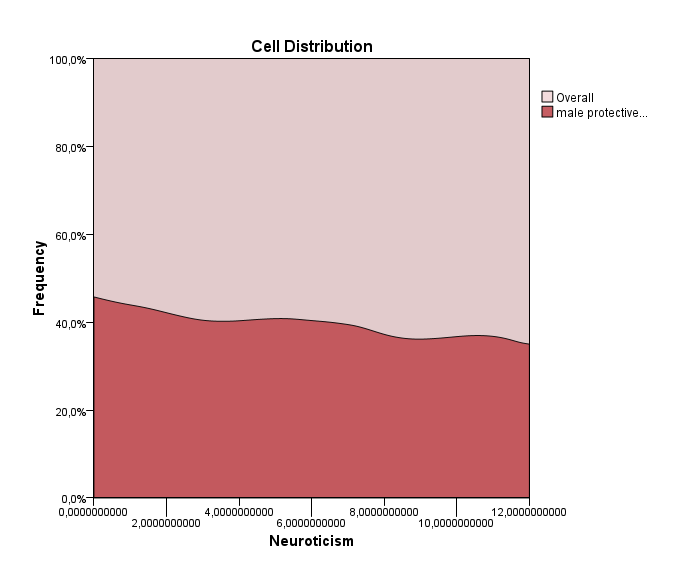 |

***Supplementary Figure 1.* Relative distribution of the clustering (input) variables in the two male clusters.** Frequency of each value can be seen in the specific cluster, relative to the overall sample of males.

| **Female risk cluster** | **Female protective cluster** |
| --- | --- |
| 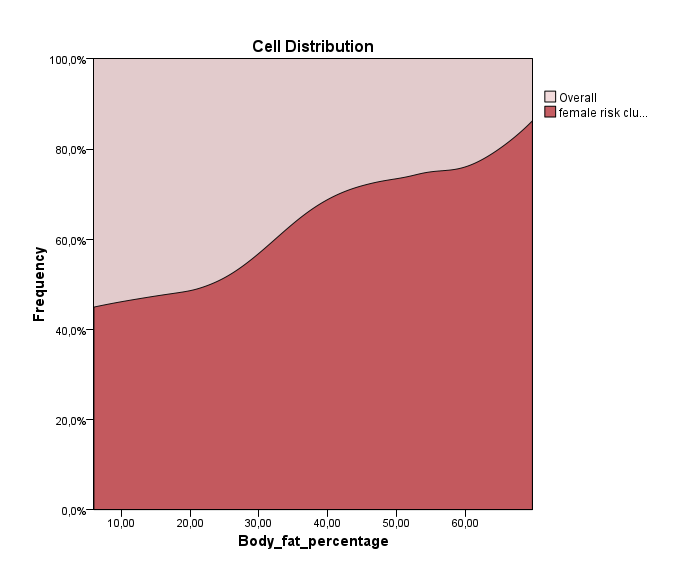 | 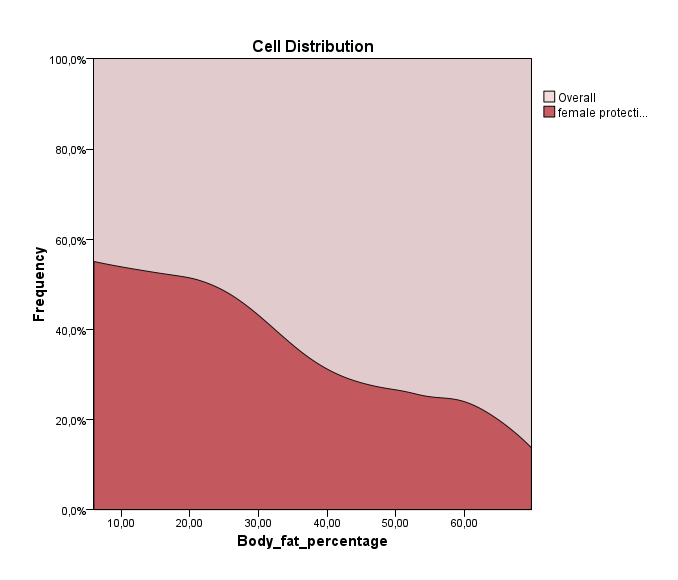 |
| 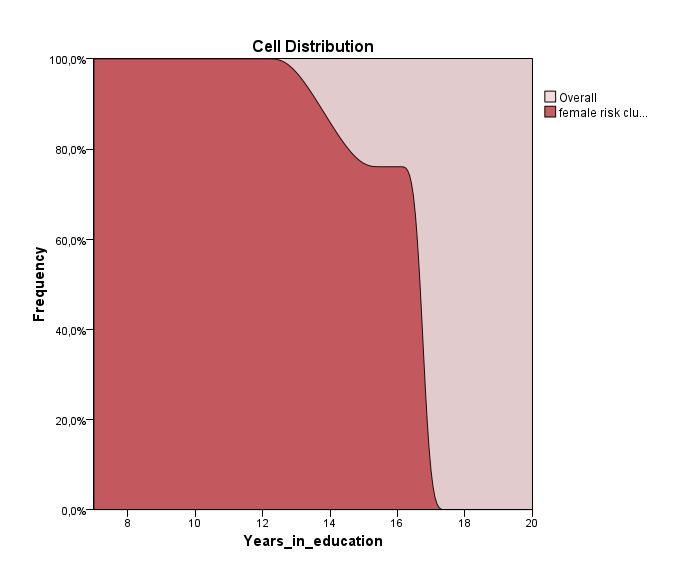 | 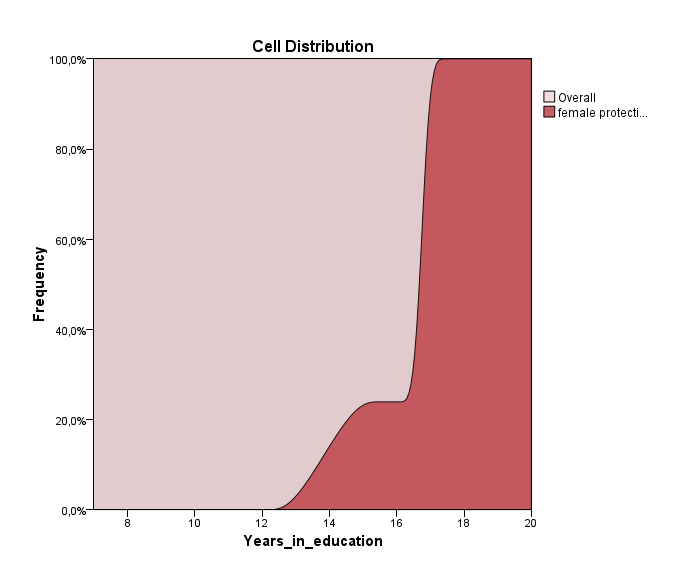 |
| 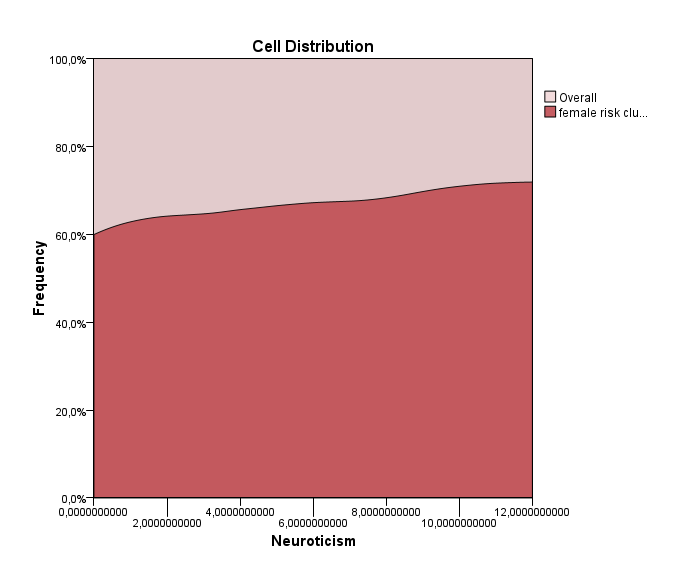 | 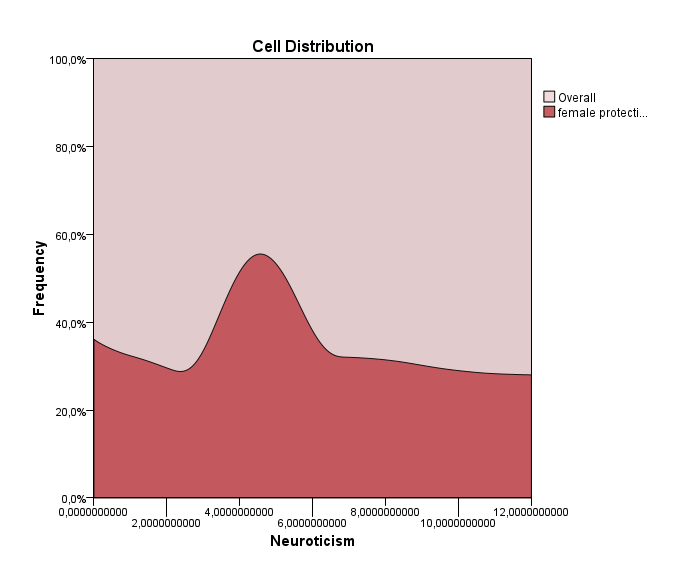 |

***Supplementary Figure 2.* Relative distribution of the clustering (input) variables in the two female clusters.** Frequency of each value can be seen in the specific cluster, relative to the overall sample of females.

The male risk cluster is slightly but significantly (t=-17.976; df=144354.723; p<0.001) older (mean age: 57.399 years) than the male protective cluster (mean age: 56.647). Similarly, the female risk cluster is also slightly but significantly (t=-56.709; df=132713.795; p<0.001) older (mean age: 57.415) than the female protective cluster (mean age: 55.214).

The male risk cluster is also slightly but significantly more depressed according to current depression score than the male protective cluster (means: 5.576 vs 5.289; t=-27.887; df=149653.255; p<0.001). Similarly, the female risk cluster is also slightly but significantly more depressed than the female protective cluster (means: 5.797 vs 5.585; t=-20.578; df=145069.153; p<0.001).
